# Naturally Derived Phenethyl Isothiocyanate Modulates Induction of Oxidative Stress via Its *N*-Acetylated Cysteine Conjugated form in Malignant Melanoma

**DOI:** 10.3390/antiox13010082

**Published:** 2024-01-08

**Authors:** Sotiris Kyriakou, Nikoletta Demosthenous, Tom Amery, Kyle J. Stewart, Paul G. Winyard, Rodrigo Franco, Aglaia Pappa, Mihalis I. Panayiotidis

**Affiliations:** 1 Department of Cancer Genetics, Therapeutics & Ultrastructural Pathology, The Cyprus Institute of Neurology & Genetics, Nicosia 2371, Cyprus; sotirisk@cing.ac.cy (S.K.); nikolettad@cing.ac.cy (N.D.); 2 The Watercress Company, Dorchester DT2 8QY, UK; tom.amery@thewatercresscompany.com; 3 Watercress Research Limited, Unit 24, De Havilland Road, Exeter EX5 2GE, UK; kyle@watercress-research.com (K.J.S.); paul@watercress-research.com (P.G.W.); 4 Redox Biology Centre, University of Nebraska-Lincoln, Lincoln, NE 68583, USA; rodrigo.franco@unl.edu; 5 Department of Veterinary Medicine & Biomedical Sciences, University of Nebraska-Lincoln, Lincoln, NE 68583, USA; 6 Department of Molecular Biology & Genetics, Democritus University of Thrace, 68100 Alexandroupolis, Greece; apappa@mbg.duth.gr

**Keywords:** watercress, isothiocyanates, phenethyl isothiocyanate, polyphenols, oxidative stress, malignant melanoma, antioxidant enzymes, mercapturic acid pathway

## Abstract

Phenethyl isothiocyanate (PEITC) is a secondary metabolic product yielded upon the hydrolysis of gluconasturtiin and it is highly accumulated in the flowers of watercress. The aim of the current study was to assess the role of a naturally derived PEITC-enriched extract in the induction of oxidative stress and to evaluate its anti-melanoma potency through the regulation of its metabolism with the concurrent production of the *N*-acetyl cysteine conjugated by-product. For this purpose, an in vitro melanoma model was utilized consisting of human primary (A375) cells as well as metastatic (COLO-679) malignant melanoma cells together with non-tumorigenic immortalized keratinocytes (HaCaT). Cytotoxicity was assessed via the Alamar Blue assay whereas the antioxidant/prooxidant activity of PEITC was determined via spectrophotometric assays. Finally, kinetic characterization of the end-product of PEITC metabolism was monitored via UPLC coupled to a tandem mass spectrometry (MS/MS). Our results indicate that although PhEF showed very minor antioxidant activity in a cell-free system, in a cell-based system, it can modulate the activity of key enzyme(s) involved in cellular antioxidant defense mechanism(s). In addition, we have shown that PhEF induces lipid and protein oxidation in a concentration-dependent manner, while its cytotoxicity is not only dependent on PEITC itself but also on its *N*-acetylated cysteine conjugated form.

## 1. Introduction

Watercress (*Nasturtium officinale*) belongs to the *Brassicaceae* family and it is a well-known aquatic plant due to its high content of phytochemicals with high nutritional impact including polyphenols (quercetin-3-*O*-rutinoside, kaempferol-3-*O*-rutinoside, isorhamnetin, protocatechuic acid and chlorogenic acid), minerals (Ca, K, Zn, Fe), vitamins (A, B, C, D and K), soluble sugars and proteins, among others [1,2,3]. One of the most valuable secondary metabolites is phenethyl isothiocyanate (PEITC). Plant tissue disruption activates endogenous myrosinase which catalyzes the bio-conversion of glucosinolates to isothiocyanates via cleavage of the thioglycosidic bond [4,5,6,7]. PEITC originates from gluconasturtiin and has been previously shown to be highly accumulated in watercress flowers while it is less abundant in leaves and stems [3]. Numerous studies have documented that PEITC can be utilized therapeutically in various human diseases including hyperglycemia, hypertension, hypercholesterolemia, bronchitis, arthritis and scurvy [8,9,10]. In addition, PEITC was shown to induce anticancer potency in various cancer types including cervical, breast, lung, prostate, melanoma and leukemia [10,11,12,13,14]. Recently, a study demonstrated that a naturally derived watercress flowers-based PEITC-enriched extract (PhEF) was capable of inducing the activation of intrinsic apoptosis via subcellular ultrastructural alterations and perturbations in Ca^2+^ efflux in malignant melanoma [15]. Furthermore, it appears that the PEITC-induced cytotoxicity was associated with its electrophilic carbon center which can react with cellular nucleophiles (e.g., thiols and amines) to form thiocarbamates/dithiocarbamates and thioureas [16,17]. In particular, it has been shown that PEITC can be conjugated with intracellular glutathione (GSH) causing its depletion thereby leading to elevated reactive oxygen species (ROS) [18,19,20,21]. Moreover, the resultant PEITC-GSH conjugate enters the mercapturic acid pathway where it is then enzymatically converted into its respective *N*-acetylated cysteine derivative (PEITC-NAC) and finally excreted via urine [22,23,24,25]. In addition, it has been suggested that the formed conjugates (mercapturic acid intermediate by-products) can modify intracellular proteins by exchanging their cysteine sulfhydryl groups with the amino or thiol groups of protein side chains causing protein oxidation resulting in elevated ROS levels [26,27]. As a result, PEITC-induced cytotoxicity is thought not to be attributed to PEITC itself but rather to its PEITC-NAC conjugated form. To this end, when human lung carcinoma (A549) cells were exposed to PEITC-NAC, apoptotic induction was observed suggesting the anti-neoplastic potency of the PEITC-NAC conjugate [28]. Similar observations were also made by other groups when dietary PEITC-NAC supplementation, in mice, inhibited prostate cancer growth by modulating the cell cycle and apoptosis [29].

The aim of the current study was to investigate the capacity of PhEF, previously characterized by our group [3,15], to cause perturbations in oxidative stress status in malignant melanoma. To this end, we have aimed to investigate the potential role of PEITC-NAC in inducing cytotoxicity to human malignant melanoma (A375, COLO-679) as opposed to neighboring, non-tumorigenic immortalized keratinocyte (HaCaT) cells. 

## 2. Materials and Methods

### 2.1. Materials 

Solvents: Methanol (LC-MS grade, purity ≥ 99.9%), water (HPLC grade), hexane (≥97%), ethyl acetate (≥99.5%) and acetonitrile (99.9%) were purchased from Honeywell, Nicosia, Cyprus. Acetic acid (purity ≥ 99.7%,) and hydrochloric acid (37%) was purchased from Sigma Aldrich, Saint Louis, MO, USA. Formic acid was purchased from Thermo Fisher Scientific, Nicosia, Cyprus. Reagents: Dimethyl sulfoxide (DMSO) was purchased from PanBiotech, Athens, Greece. *N*-acetyl cysteine (NAC), thiobarbituric acid, magnesium sulphate, guanidine hydrochloride and resazurin sodium salt were purchased from Fluorochem, Derbyshire, UK. 2,4-dinitro phenyl hydrazine (2,4-DNPH) and TBA malondialdehyde (MDA) standard were purchased from Cayman Chemicals, Ann Arbor, MI, USA. Glutathione (GSH), NADPH, Nitrotetrazolium Blue, ethylenediaminetetraacetic acid (EDTA), ethacrynic acid, PEITC, hydrogen peroxide (30% aqueous solution), xanthine, xanthine oxidase, glutathione reductase, cumene hydroperoxide (80%), perchloric acid (70%), sodium carbonate, sodium hydroxide and Ellman’s reagent were purchased from Sigma Aldrich, Nicosia, Cyprus. Assay kits: The bicinchoninic acid (BCA) protein assay kit was purchased from Thermo Scientific, Waltham, MA, USA, 2,2-azinobis (3-ethyl-benzothiazoline-6-sulfonic acid) (ABTS^●+^), 2,2-diphenyl-1-picrylhydrazyl (DPPH^●^), Ferric reducing antioxidant power (FRAP) were purchased from Bioquochem, Asturia, Spain. The TBARS and protein carbonyl colorimetric assay kits were purchased from Cambridge Bioscience Ltd., Cambridge, UK. Cell culture reagents: Dulbecco’s Modified Eagles Medium (DMEM) high glucose media, Roswell Park Memorial Institute (RPMI) 1640, Fetal Bovine Serum (FBS), *L*-glutamine, Pen/Strep (100 U/mL penicillin/100 μg/mL streptomycin), trypsin-EDTA (100×) and phosphate buffer saline (PBS; *w*/*o* calcium and magnesium) were purchased from BIOSERA, Athens, Greece). 

### 2.2. Synthesis of N_α_-Acetyl-S-(N-phenethylthiocarbamoyl)-glutathione

In a stirring solution of NAC (1.0 g, 6.13 mmol, 1.2 eq) in methanol (5 mL), phenethyl isothiocyanate (PEITC) (5.1 mmol, 1 eq) was added. The reaction mixture was allowed to be stirred at room temperature (RT) until the formation of a heterogenous solution and then it was heated at 50 °C for a further 2 h. Upon completion of the reaction, methanol was removed under reduced pressure and the resulting residue was taken up in an aqueous solution of sodium hydroxide (1 M) and washed once with *n*-hexane. The pH of the aqueous phase was adjusted to 2 by the addition of concentrated hydrochloric acid and the crude product was extracted with ethyl acetate. The companied extracts were washed with excess of water, dried over magnesium sulphate and concentrated under reduced pressure affording the title product as a pale–yellow oil (0.39 g, 2.86 mmol, 56%). NMR Spectroscopy was performed using a Bruker Avance spectrometer at frequencies of 500 MHz for ^1^H-NMR, 100 MHz for ^13^C-NMR.The produced spectra were analyzed and processed with TopSpin (version 3.6.5) software. Chemical shifts were recorded as parts per million (ppm) with tetramethylsilane (TMS) as the internal standard. The solvent included deuterated dimethyl sulfoxide (DMSO-*d_6_*). Chemical shifts were observed with integrals, splitting and *J* values, multiplicity of the signals were recorded as singlet (s), doublet (d), triplet (t), multiplet (m) and broad (br). In addition, the multiplicities were recorded: ^1^H-NMR (500 MHz, DMSO-*d_6_*); δ_H_: 1.84 (s, 3H, COCH_3_), 2.88 (t, *J* = 8.8 Hz, 2H, -SCH_2_CH-), 3.30 (t, *J* = 6.8 Hz, 2H, -CH_2_CH_2_NH-), 3.75 (t, *J* = 6.8Hz, 2H, -CH_2_CH_2_NH-), 4.37–4.37 (m, 1H, -NHCHCOOH), 7.21–7.30 (m, 5H, *Ar*), 8.31 (d, *J* = 8.2 Hz, 1H, -CH_2_NHS-), 10.16 (s, 1H, -CHNHCO-), 12.86 (s, br, -COOH) ppm;^13^C-NMR (126 MHz, DMSO-*d_6_*); δ_C_: 22.2 (-NHCOCH_3_), 33.1 (-SCH_2_-), 35.3 (-CH_2_CH_2_NH-), 47.9 (-CH_2_CH_2_NH-), 52.0 (-CH-), 126.0 (*Ar*), 128.2 (*Ar*) 128.4 (*Ar*), 138.6 (*Ar*),169.1 (-COCH_3_),171.7 (-COOH) 195.2 (-CS-) ppm. 

### 2.3. Processing and Storage of Plant Material 

Watercress flower samples were provided by The Watercress Company, Dorchester, Dorset, UK. All samples were maintained at −20 °C until further use. Watercress flowers were immersed in liquid nitrogen prior to drying them in a freeze-drier (Christ Alpha 1-4, LSC Basics, Osterode, Germany) at −55 °C, 0.05 mbar for 96 h. The de-hydrated parts were then re-immersed in liquid nitrogen and grinded to a fine powder using a domestic blender. Powdered samples were kept at −20 °C in a sealed bag protected from air, humidity and light until further use.

### 2.4. Extraction of Phenethyl Isothiocyanate-Enriched Fraction (PhEF)

The extraction of PEITC was performed as previously described [3]. Briefly, 5.0 g of lyophilized watercress flowers were suspended in phosphate-buffered saline (PBS; pH 7) (150 mL) containing a catalytic amount of ascorbic acid. The formed suspension was incubated at 37 °C under continuous stirring for 1 h in order to stimulate the hydrolysis of glucosinolates. The resulting solution (150 mL) was filtered over Whatman filter paper (pore size: 4.0–12 μm) and the filtrates were subsequently extracted by convectional liquid–liquid extraction using *n*-hexanes (3 washes × 150 mL each). Isolation and drying of the organic phase over magnesium sulphate followed by concentration to dryness under reduced pressure yielded a phenethyl isothiocyanate-enriched fraction as a viscous oil (2.1 g, 1.91 mL, yield: 42%). The yield was calculated based on the mass of dry extract.

### 2.5. Extraction of Polyphenols-Enriched Fraction (PoEF)

The extraction of polyphenols was performed as previously described [3]. Briefly, 5.0 g of watercress flowers was exhaustively macerated at 80 °C in aqueous methanol (80% *v*/*v*) (100 mL) for 48 h. The resulting suspension was filtered through Whatman filter paper (pore size: 4.0–12.0 μm). The process was repeated twice and the combined solutions were concentrated under reduced pressure affording the PoEF as a brown paste.

### 2.6. Determination of N_α_-Acetyl-S-(N-phenethylthiocarbamoyl)-glutathione Accumulation 

The concentration of *N*_α_-acetyl-*S*-(*N*-phenethylthiocarbamoyl)-glutathione (PEITC-NAC conjugate) was assayed intracellularly (in A375 cells) and extracellularly (in serum-free medium to prevent adduct formation between PEITC and thiol groups found in serum). Briefly, A375 cells were exposed to PhEF with or without the addition of either ethacrynic acid (30 μM) or *N*-ethylmaleimide (25 μΜ) at various time points (0–48 h). Cells were then trypsinized and washed with PBS with the resultant pellet being de-proteinized with perchloric acid (0.6 M). The formed suspension was centrifuged at 3000× *g* for 3 min. The supernatant was transferred in clean tubes and spiked with the pre-synthesized adduct (0.5 mM). The solution was allowed to stand at RT for 30 min and then neutralized by sodium carbonate (2 M). The final solution was passed through 0.2 μm mix cellulose ester filters and analyzed immediately by UPLC-MS/MS. For assaying the extracellular content, all conditions were as described above except that de-proteinization of the culture medium was performed with 1.2 M of perchloric acid.

### 2.7. Liquid Chromatography (LC) and Tandem Mass Spectrometry (MS/MS) Conditions

For the detection and quantification *N*_α_-acetyl-*S*-(*N*-phenethylthiocarbamoyl)-glutathione, a Waters ACQUITY UPLC system (Waters Corp., Milford, MA, USA) was used. The chromatographic separation was performed on an ACQUITY UPLC BEH C18 (100 × 2.1 mm, particle size: 1.7 μm) column (Waters Corp., Milford, MA, USA), heated at 30 °C. The mobile phase consisted of a solution of acetonitrile (eluent A) and formic acid 0.1% (*v*/*v*) (eluent B). A flowrate of 300 μL/min was used and the applied gradient conditions consisted of 6.7 min (100% A), 7.5 min (98% A), 10 min (96% A), 11.5 min (94% A), 12.5 min (92% A), 13 min (90% A), 15 min (88% A), 20 min (85% A), 25 min (80% A), 30 min (70% A), 35 min (65% A), 40 min (60% A), 42 min (50% A), 43.5 min (0% A) and 52 min (100% A).

For the MS/MS experiments, a Xevo Triple Quadrupole (TQD) Mass detector (Waters Corp., Milford, MA, USA) was operated in positive ionization mode (ESI+). Detection of the analytes was performed utilizing the collision voltage (MS1), whereas the quantitative analysis was accomplished using selected multiple reaction monitoring (MRM) mode. The MRM conditions were optimized for the synthesized standard, by MS manual tuning prior to sample analysis at a concentration of 1 ppm. To acquire maximum signals, the optimized tuning parameters were as follows: capillary voltage: 2.5–3.0 kV; cone voltage: 36 V; source temperature: 150 °C; dissolvation temperature: 500 °C; source dissolvating gas flow: 1000 L/h and gas flow: 20 L/h. High-purity nitrogen gas was used as the drying and nebulizing gas, whereas ultrahigh-purity argon was used as a collision gas. Data acquisition and processing were performed on MassLynx software (version 4.1). 

### 2.8. Determination of Cell-Free Antioxidant Activity Levels 

Cell-free antioxidant activity of PhEF and PoEF was estimated by the ABTS^●+^, DPPH^●^ and FRAP assay kits according to the manufactures’ recommendations. The results were calculated as % Radical (Cation) Inhibition according to the following equation:

(1) $$\%\;\mathrm{Radical}\;\mathrm{Inhibition}=\left(\frac{1-A_{f}}{A_{0}}\right)\times 100$$

where A_0_ is the absorbance of the non-inhibited radical cation whereas A*_f_* is the absorbance recorded at 715 nm and 517 nm for ABTS^●+^ and DPPH^●^ respectively. Values are expressed as half-maximal inhibitory concentration (IC_50_) in % *v*/*v*. For the FRAP assay, the potency of PhEF and PoEF was expressed as mmols inhibited ferric cations (Fe^2+^)/g of dry extract.

### 2.9. Cell Lines

The human malignant melanoma (A375) and (COLO-679) cell lines were purchased from the American Type Culture Collection (ATCC) Manassas, VA, USA and Deutsche Sammlung von Microorganismen und Zellkulturen (DSMZ) Braunschweig, Germany, respectively. The human immortalized keratinocyte (HaCaT) cell line was kindly provided by Dr Sharon Broby (Dermal Toxicology and Effects Group; Centre for Radiation, Chemical and Environmental Hazards; Public Health England, Chilton, UK). A375 cells were cultured in DMEM high glucose whereas COLO-679 cells were grown in RPMI media. Both types of culture media were supplemented with 10% FBS, 2 mM *L*-glutamine and 1% pen/strep (100 U/mL penicillin/100 μg/mL streptomycin). Cells were grown in a humidified incubator at 37 °C and 5% CO_2_, as monolayers and sub-cultured at 80–90% confluency. 

### 2.10. Determination of Intracellular Superoxide Dismutase (SOD), Catalase (CAT), Glutathione Peroxidase (GPx) and Glutathione Reductase (GR) as Well as Glutathione S-Transferase (GST) Activity Levels 

A375 cells (at a density of 1.5 × 10^6^ cells/ plate) were seeded in 100 mm plates and incubated overnight. The next day, cells were treated with either DMSO (0.1% *v*/*v*) or PhEF (0.75–2.5% *v*/*v*) or PoEF (0.75–2.5% *v*/*v*) and incubated for a further 24 h. Cells were harvested in trypsin-EDTA, washed with PBS and centrifuged at 2000 rpm for 3 min. Pellets were then resuspended in ice-cold PBS for the determination of SOD, GR, GST, GPx and CAT activities. For the GSH assay, the pellet was re-dissolved in 5% ice-cold sulfosalicylic acid (300 μL) and then cells were lysed by sonication at a 20 s on and 10 s off cycle. Following centrifugation at 15,000× *g* at 4 °C the supernatants were collected on fresh tubes and stored at −80 °C until used. The protein content of each extract was determined via BCA assay kit according to the manufacture instructions. 

For SOD activity, results were obtained by adopting a previously published procedure with some modifications [30]. In brief, cell lysates (20 μL) were mixed with 50 mM sodium carbonate buffer (160 μL, pH 10) containing 3 mM EDTA, 3 mM xanthine, 1.5 mg/mL bovine serum albumin (BSA) and 0.75 mM of nitrotetrazolium blue. The reaction was initiated by the addition of xanthine oxidase (20 μL, 13.2 U/mL). After 30 min of incubation, the reaction was quenched by the addition of 6 M copper chloride (CuCl_2_) (200 μL). The absorbance of formazan was monitored at 560 nm on a microplate reader (LT4500, Labtech, UK). Results were expressed as %SOD activity (of control)/mg of protein. 

For CAT activity, results were obtained as previously described [31,32]. Cell lysates (200 μL) were suspended in potassium phosphate buffer (1790 μL, 50 mM, pH 7.0). Then, hydrogen peroxide (H_2_O_2_; 10 μL, 30%) was added and its degradation by catalase was monitored at 240 nm on a a microplate reader (LT4500, Labtech, UK). Results were expressed as %CAT activity (of control)/mg of protein. 

For GPx activity, results were obtained as previously described [33]. Briefly, cell lysates (20 μL) were mixed with potassium phosphate buffer (187.5 μL, 50 mM, pH 7.0) and NADPH (4 mM) (12.5 μL) while the resulting solution was incubated at RT for 5 min. The reaction was initiated by the addition of 0.15% cumene hydroperoxide (100 μL). The rate of NADPH consumption was monitored at 340 nm on a a microplate reader (LT4500, Labtech, UK). Results were expressed as %GPx activity (of control)/mg of protein.

For GR activity, results were obtained by mixing cell extracts (20 μL) with phosphate buffer (120 μL, 50 mM, pH 7.0), 2 mM oxidized glutathione (GSSH) (40 μL) and 3 mM nitrotetrazolium blue (10 μL). Then, 2 mM NADPH (10 μL) was added and the reaction was monitored for a period of 5 min and measurements were collected every 30 s. The absorbance of formazan was measured at 560 nm on a microplate reader (LT4500, Labtech, UK). Results were expressed as %GR activity (of control)/mg of protein.

For GST activity, results were obtained by mixing PBS (120 μL) with GSH (200 mM) (50 μL), cell lysates (20 μL) and 1-chloro-2,4-dinitrobenzene (CDNB) (100 mM) (10 μL). The absorbance of the resulting solution was monitored at 340 nm, every 30 s, for a period of 5 min. The results were expressed as %GST activity (of control)/mg of protein. 

### 2.11. Determination of Total Glutathione (GSH) Content

For the determination of intracellular GSH content, 0.35 mM NADPH (60 μL) was mixed with 6 mM of Ellman’s reagent (5,5′-Dithiobis (2-nitrobenzoic acid; DTNB) (10 μL) and either cell (20 μL) lysates or GSH standard. All solutions were prepared in sodium phosphate buffer (100 μL, 125 mM, pH 7.5) containing 6.3 mM EDTA. The reaction was initiated by the addition of GR (10 μL, 5 IU/ mL). The rate of reaction was monitored spectrophotometrically (LT4500, Labtech, Heathfield, UK) at 412 nm. The results were expressed as GSH (mmol)/mg of protein (% of control).

### 2.12. Determination of Cell Viability

The Alamar Blue assay was utilized. Briefly, cells were seeded in 96-well plates and incubated overnight prior to exposure. A375 densities were 8000, 4000 and 2000 cells/well, whereas HaCaT and COLO-679 densities were 10,000, 5000 and 2500 cells/ well. On the following day, cells were exposed to a range of concentrations of either PhEF or PoEF (in 0.1% *v*/*v* DMSO) for 0–48 h in the presence or absence of ethacrynic acid and/or *N*-ethylmaleimide. For control conditions, cells were incubated with complete medium only or 0.1% *v*/*v* DMSO. At indicated time points, resazurin (dissolved in PBS at 1 mg/mL final concentration) was added and further incubated for 4 h at 37 °C. Absorbance was recorded at 570 nm and 590 nm (reference wavelength) using a microplate reader (LT4500, Labtech, UK). Cell viability was expressed as a percentage of control cells.

### 2.13. Determination of Lipid Peroxidation and Protein Carbonyl Contents 

A375, COLO-679 and HaCaT cells were seeded and incubated overnight. The next day, cells were treated with either DMSO (0.1% *v*/*v*) or PhEF (0–2.5% *v*/*v*) or PoEF (0–2.5% *v*/*v*) for 24 h. After trypsinization, pellets were collected, re-suspended in PBS and sonicated. For the determination of the lipid peroxidation content, the entire cell suspension was further diluted with 4% *v*/*v* acetic acid solution containing 8.0% thiobarbituric acid (TBA). The final mixture was heated at 95 °C for 1 h and centrifuged at 3000 rpm for 2 min. The TBARS assay kit was utilized for the determination of malondialdehyde (MDA) content according to the manufacture’s protocol. For the determination of protein carbonyl content, cells were trypsinized and pellets were collected, re-suspended in PBS (supplemented with 1 mM EDTA) and sonicated. The protein carbonyl colorimetric assay kit was utilized according to the manufacture’s protocol.

### 2.14. Statistical Analyses 

Data were expressed as mean values ± standard deviation (SD) and comparisons were made between control and treated groups. Statistical analyses were performed by one-way ANOVA with Tukey’s test for multiple comparisons using the GraphPad Prism 6 software. Statistical significance was set at *p* < 0.05, *p* < 0.01, *p* < 0.001 and *p* < 0.0001.

## 3. Results

### 3.1. Synthesis of Phenethyl Isothiocyanate-N-Acetyl Cysteine Adduct 

The synthesis of *N*_α_-*acetyl-S*-(*N*-phenethylthiocarbamoyl)-glutathione (PEITC-NAC conjugate; the end-product of PEITC metabolism through the mercapturic acid pathway) produced a good yield, while its purity was assessed via UPLC-MS/MS (Figure S1), ^1^H- (Figure S2A) and ^13^C-NMR (Figure S2B).

### 3.2. Standardization of UPLC and MS Conditions

Quantification of the PEITC-NAC conjugate adduct, both intracellular and extracellularly was performed by utilizing the synthesized product as a reference standard. For quantification purposes, we employed UPLC tandem mass spectrometry (UPLC-MS/MS) operating in the MRM transitions mode using the parameters; cone voltage and collision energy as obtained from manual tuning (Table S1, Figure S3). The combinations of the mobile phase, elution mode, flow rate and column that have been used in the current study were chosen in order to acquire the optimal signal for the analyte. Various solvent combinations were utilized including those of methanol/water and acetonitrile/water in different ratios. However, none of these yielded peaks of optimum shape and sharpness. Improvement of peak shape and symmetry was achieved by the acidification of water with formic acid (0.1% *v*/*v*). For the ionization of the PEITC-NAC adduct, the electron spray ionization (ESI) was operated in the positive mode (ESI^+^).

### 3.3. Method Validation, Linearity, Precision and Reproducibility of Methodology

The analytical method was validated according to the guidelines listed in the European Medicines Agency. Namely, linearity, limit of quantification (*L*o*Q*), limits of detection (*L*o*D*) and precision were determined (Table S2). The generated calibration curve of the synthesized standard was plotted using the linear regression equation of the peak intensity area versus various concentrations (ranging from 0.21–45 nM) (Figure S4). The PEITC-NAC adduct showed good linearity, whereas the respective correlation coefficient (R^2^) was >0.99 (Table S2). Limit of detection and quantification values were calculated based on the signal-to-noise ratio (*S/N*) which was set at 3 and 10, respectively. The *L*o*D* and *L*o*Q* values were shown to be equal to 0.21 and 6.93 nM, respectively (Table S2). Precision of the method was assessed by means of calculating the percent of relative standard deviation (% RSD). For this purpose, six replicated samples at the same concentration of the analyte were analyzed within one day and within six consecutive days for the estimation of intra- and inter-day precision; the percentages were found to be 0.96 and 2.56%, respectively (Table S2). Eventually, the reproducibility of the UPLC-ESI(+)-MS/MS was evaluated by determining the % recovery based on the quantification procedure (Table S2). For this purpose, each control cell extract was spiked with a known concentration of the adduct and the % recovery was calculated according to Equation (2) where A is the final concentration detected, A_0_ is the initial concentration of the standard and A_a_ is the concentration of the added adduct.

(2) $$\%\;\mathrm{Recovery}=\left[\frac{A-A_{0}}{A_{a}}\right]\times 100$$

Overall, the average %recovery was found to be equal to 98.8%, suggesting the accuracy and reproducibility of our methodology. 

### 3.4. Evaluation of Cell-Free Antioxidant Activity 

**Figure 1:**
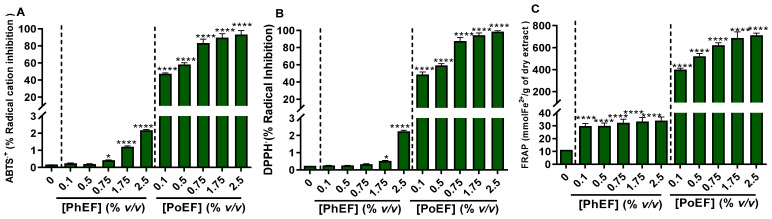
Cell-free antioxidant capacity of PhEF and PoEF as determined by (**A**) ABTS^●+^, (**B**) DPPH^●^ and (**C**) FRAP assays. Data shown are the means ± SEM of three independent experiments. Statistical comparisons were conducted between control and treated samples. Asterisk(s) * denote(s) statistical significance at *p* ≤ 0.05, whereas **** at *p* ≤ 0.0001.

Next, the cell-free antioxidant activity was evaluated by means of investigating the ability of PhEF and PoEF to quench the free radical of ABTS^●+^ (Figure 1A, Table 1) and DPPH^●^ (Figure 1B, Table 1) as well as ferrous (Fe^2+^) ions (Figure 1C, Table 1). 

As shown in Figure 1, PhEF does not exhibit significant potency towards radical quenching nor towards chelating Fe^2+^ ions, thus suggesting the inability of PEITC to act as an antioxidant compared to PoEF (indicated by its low IC_50_ values; Table 1). More specifically, at the highest concentration of the extract (2.5% *v*/*v*), only 2% and 2.5% of radical scavenging in DPPH^●^ and ABTS^●+^, respectively, was observed, denoting the poor antioxidant potency of PhEF. On the contrary, under the same concentration, PoEF, achieved an almost 100% radical scavenging both in DPPH^●^ and ABTS^●+^.

### 3.5. Evaluation of Intracellular Antioxidant Capacity 

**Figure 2:**
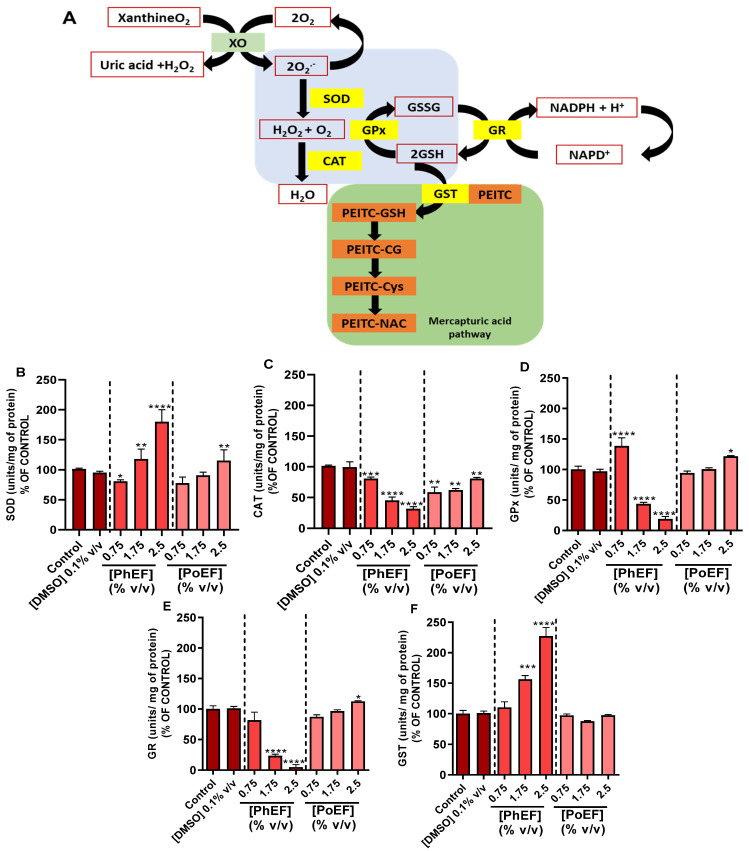
The ability of PhEF to modulate the activity of (**A**) major antioxidant enzymes, (**B**) SOD, (**C**) CAT, (**D**) GPx, (**E**) GR and (**F**) GST. Data shown are means ± SEM of three independent experiments. Statistical comparisons were conducted between control and treated samples. Asterisk(s) *, **, *** and **** denote(s) statistical significance at *p* ≤ 0.05, *p* ≤ 0.01, *p* ≤ 0.001 and *p* ≤ 0.0001, respectively.

The increased production of cellular ROS includes, but is not limited to, superoxide radicals and hydroperoxides, which are known to perpetuate cellular damage. Consequently, they are metabolized by corresponding intracellular antioxidant enzymes including SOD and CAT. In addition, metabolism of H_2_O_2_ is also performed by the action of GPx utilizing glutathione as a substrate. Finally, the resultant oxidized glutathione is recycled back to its reduced form by the action of GR (Figure 2A).

In order to elucidate a possible mechanism for the anticancer capacity of the PhEF, its ability to modulate oxidative stress via intracellular ROS production was investigated. For this reason, the activity of SOD, CAT, GPx, GR and GST to metabolize ROS was studied. To this end, A375 cells were subjected to PhEF at non-toxic (below EC_50_; 0.75% *v*/*v*), sub-toxic (at EC_50_; 1.75% *v*/*v*) and toxic (above EC_50_; 2.5% *v*/*v*) concentrations as previously characterized by our group [15]. Overall, it was shown that SOD activity is proportional to the concentration of both PhEF and PoEF since the highest activity was noticed at their highest concentrations (Figure 2B). On the contrary, the activity of CAT in PhEF-subjected cells was decreasing as the PhEF concentration was increasing, while an exactly opposite pattern was observed in PoEF-subjected cells. In either way, CAT activity levels were retained at control values even at both the extracts’ highest concentrations (Figure 2C). In regard to the role of the enzymes involved in the recycling of GSH, exposure to PhEF significantly reduced the activities of GPx (Figure 2D) and GR (Figure 2E), whereas exposure to PoEF retained their enzymatic levels unaffected. Finally, the activity of GST (the primary enzyme involved in the conjugation of GSH with PEITC [34]) was shown to be significantly increased upon exposure to PhEF whereas under exposure to PoEF remained unaffected and at control levels (Figure 2F). Overall, it can be observed that under the non-toxic concentration of the extract (0.75% *v*/*v*), the activity of the above-mentioned antioxidant enzymes was slightly altered. However, as the concentration of the extract increased, their activity was further altered.

### 3.6. The Effect of PhEF in Lipid and Protein Oxidation

**Figure 3:**
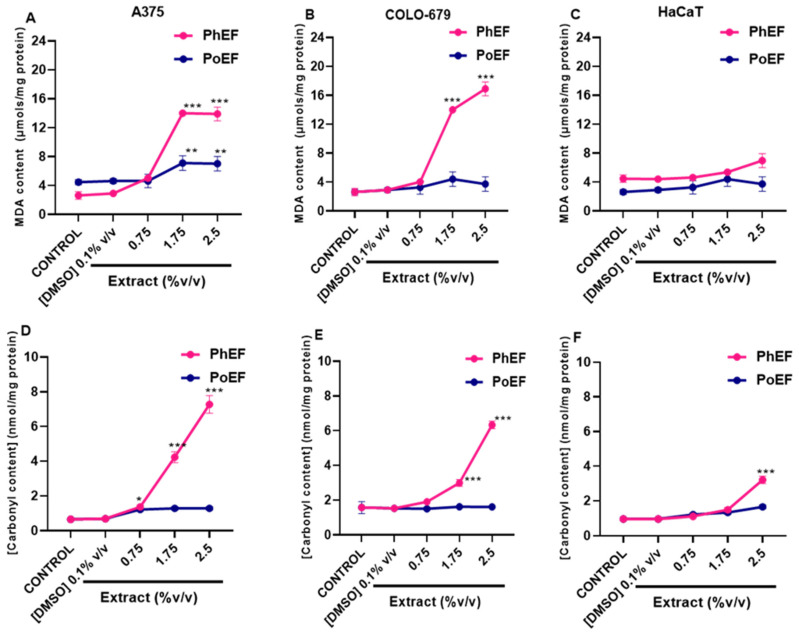
The effect of PhEF as an inducer of lipid and protein oxidation in A375, COLO-679 and HaCaT cells. The MDA and carbonyl contents were recorded, under various concentrations of PhEF, in A375 (**A**,**D**), COLO-679 (**B**,**E**) and HaCaT (**C**,**F**) cells. PoEF (0–2.5% *v*/*v*) was utilized as a negative control. Data shown are means ± SEM of three independent experiments. Statistical comparisons were conducted between control and treated samples. Asterisk(s) *, ** and *** denote(s) statistical significance at *p* ≤ 0.05, *p* ≤ 0.01 and *p* ≤ 0.001 relative to corresponding controls (DMSO 0.1% *v*/*v*).

Next, we sought to determine the effect of PhEF on biomarkers of oxidative stress generation, namely malondialdehyde (MDA; indicative of lipid peroxidation) and total protein carbonylation content. To this end, PoEF was employed as a negative control (Figure 3).

Our results suggest that formation of both MDA and carbonylated protein levels occurred in a concentration-depended manner. Specifically, at non-toxic concentrations (below EC_50_ for both extracts), there was no marked oxidation of neither lipids nor proteins in A375 (Figure 3A,D), COLO-679 (Figure 3B,E) and HaCaT (Figure 3C,F) cells. On the contrary, at sub-toxic (at EC_50_) and toxic (above EC_50_) concentrations, of both extracts, the formation of MDA and carbonylated proteins increased significantly only in PhEF- but not in PoEF-subjected A375 (Figure 3A,D) and COLO-679 (Figure 3B,E) cells, respectively. Not surprisingly, in HaCaT cells, the levels of both MDA and protein carbonylation remained at control levels with the exception of a significant elevation of protein carbonylation at toxic levels of PhEF-subjected cells (Figure 3C,F).

### 3.7. Kinetic Determination of PEITC-NAC Conjugate Formation 

**Figure 4:**
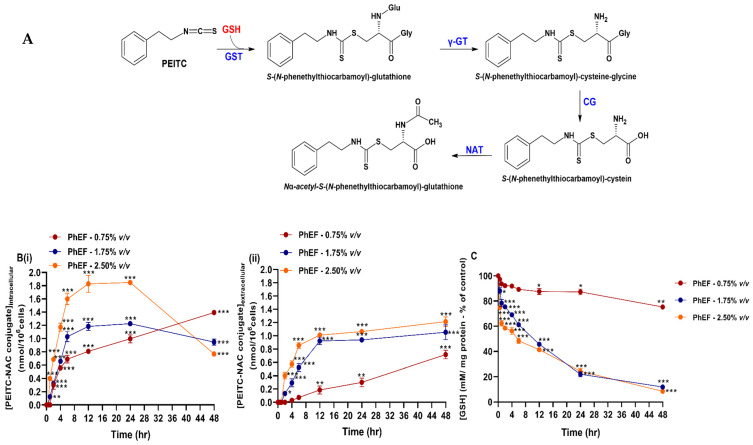
Kinetic characterization of *N*_a_-acetyl-S-(*N*-phenethylthiocarbamoyl)-glutathione [phenethyl isothiocyanate–*N*-acetyl cysteine (PEITC-NAC) conjugate] formation upon exposure to PhEF. The biosynthesis of PEITC-NAC conjugate (**A**) was measured intracellularly (**B**) (**i**) and in the culturing medium (**B**) (**ii**) at various concentrations (0.75–2.5% *v*/*v*) of PhEF over 0.5–48 h of exposure. Levels of cellular GSH were also measured at the indicated time points (**C**). Data are expressed as means ± SEM of three independent experiments. Statistical significance is indicated by * at *p* ≤ 0.05, ** at *p* ≤ 0.01 and *** at *p* < 0.001 relative to corresponding controls (DMSO 0.1% *v*/*v*).

A375 cells were subjected to various concentrations of PhEF after which cells were harvested, at indicated time points, and then spiked with the synthesized standard in order to determine the concentration profile of the mercapturic acid pathway end-product (Figure 4A). In addition, the concentration of the formed conjugated adduct was also measured in the extracellular space (serum-free medium). The results revealed that the formation of the PEITC-NAC conjugate was first noticed at 2 h post-treatment, regardless of the concentration of the extract [Figure 4B(i)] and its concentration increased (up to 6 h) suggesting of a linear trend. Not surprisingly, the highest concentration of the adduct was yielded in cells subjected to 2.5% *v*/*v* PhEF, whereas the lowest concentration was in cells subjected to 0.75% *v*/*v* [Figure 4B(i)]. In cells subjected to either 1.75% or 2.5% *v*/*v*, a plateau stage was observed at 6–24 h, whereas a marked decrease in the conjugate production was noticed at 48 h. On the contrary, in A375 cells subjected to 0.75% *v*/*v* of extract, the adduct formation was increasingly steady until the end of monitoring time course [Figure 4B(i)]. Additionally, the maximum concentration of the conjugated adduct accumulated in the extracellular space was shown to be in conditions where cells were subjected to either 1.75 or 2.5% *v*/*v* of PhEF. This time point was recorded as early as 12 h followed by a plateau stage of up to 48 h [Figure 4B(ii)]. In the case of cells subjected to 0.75% *v*/*v* PhEF, the PEITC-NAC concentration was increasing linearly until the end of the monitoring period [Figure 4B(ii)]. Furthermore, GSH levels were decreased in an analogous concentration and time dependence (Figure 4C). 

**Figure 5:**
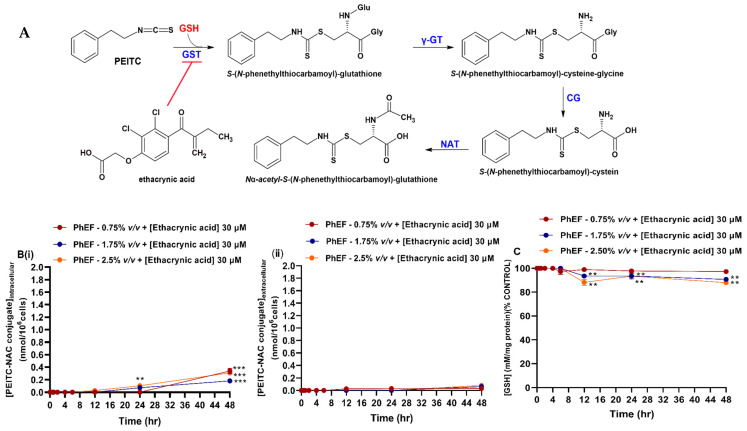
Kinetic characterization of *N*_a_-acetyl-*S*-(*N*-phenethylthiocarbamoyl)-glutathione [phenethyl isothiocyanate-*N*-acetyl cysteine (PEITC-NAC) conjugate] formation upon exposure to PhEF and ethacrynic acid. The biosynthesis of PEITC-NAC conjugate (**A**) was measured intracellularly (**B**) (**i**) and in the culturing medium (**B**) (**ii**) after 2 h of pre-treatment with ethacrynic acid (30 μM) before treatment with PhEF at various concentration (0.75–2.5% *v*/*v*) over 0.5–48 h of exposure. Levels of cellular GSH were also measured at the indicated time points (**C**). Data are expressed as means ± SEM of three independent experiments. Statistical significance is indicated by ** at *p* ≤ 0.01 and *** at *p* < 0.001 relative to corresponding controls (DMSO 0.1% *v*/*v*).

In addition, a reversible inhibitor of GST (ethacrynic acid) was utilized (Figure 5A) as previously described by others [35,36,37]. Finally, cells pre-treated for 4 h to 30 μΜ of ethacrynic acid, followed by exposure to PhEF extract (0.75–2.5% *v*/*v*) for another 0.5–48 h, provided optimized experimental conditions in the context of not being associated with the induction of any cytotoxicity (Figure S5A, Table S3). Overall, ethacrynic acid-induced inhibition of GST prevented the formation of PEITC-GSH and consequently the detection of PEITC-NAC until 24 h during which the adduct began to be produced with the effect being slightly intensified at 48 h (Figure 5B(i)). On the other hand, in the extracellular space, PEITC-NAC remained almost undetected even after 48 h [Figure 5B(ii)]. Last, GSH levels were minimally affected (at any time of exposure), suggesting prevention of GSH conjugation (Figure 5C).

**Figure 6:**
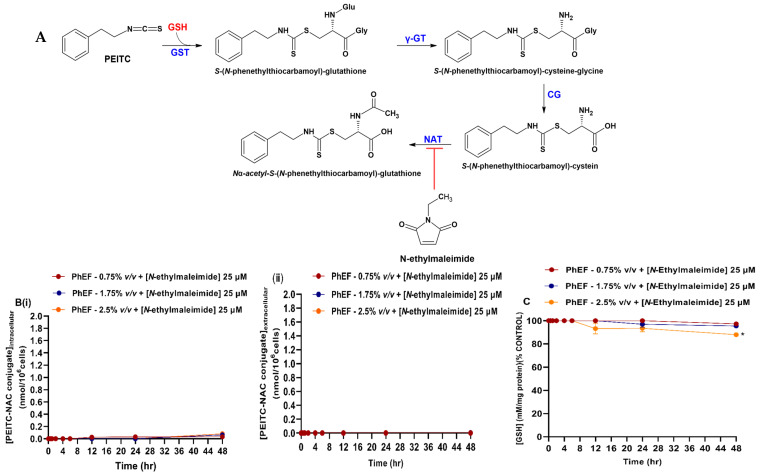
Kinetic characterization of *N*_a_-acetyl-*S*-(*N*-phenethylthiocarbamoyl)-glutathione [phenethyl isothiocyanate-*N*-acetyl cysteine (PEITC-NAC) conjugate] formation upon exposure to PhEF and *N*-ethylmaleimide. The biosynthesis of PEITC-NAC conjugate (**A**) was measured intracellularly (**B**) (**i**) and in the culture medium (**B**) (**ii**) after 2 h pre-treatment with *N*-ethylmaleimide (25 μM) before treatment with PhEF at various concentrations (0.75–2.5% *v*/*v*) over 0.5–48 h of exposure. Levels of cellular GSH were also measured at the indicated time points (**C**). Data are expressed as means ± SEM of three independent experiments. Statistical significance is indicated by * at *p* ≤ 0.05, relative to corresponding controls (DMSO 0.1% *v*/*v*).

Finally, *N*-ethylmaleimide (an inhibitor of NAT) was utilized (Figure 6A) as suggested by others [38,39,40]. Briefly, a concentration of 25 μΜ was determined to be optimal in completely abolishing the effect of PhEF (Figure S5B, Table S3). Overall, the inhibition of NAT caused complete inhibition of the adduct formation as it was neither detected intracellularly [Figure 6B(i)] nor in the culture medium [Figure 6B(ii)]. Finally, GSH levels were retained at control levels (Figure 6C).

### 3.8. The Effect of Mercapturic Acid Inhibition on Cell Viability

**Figure 7:**
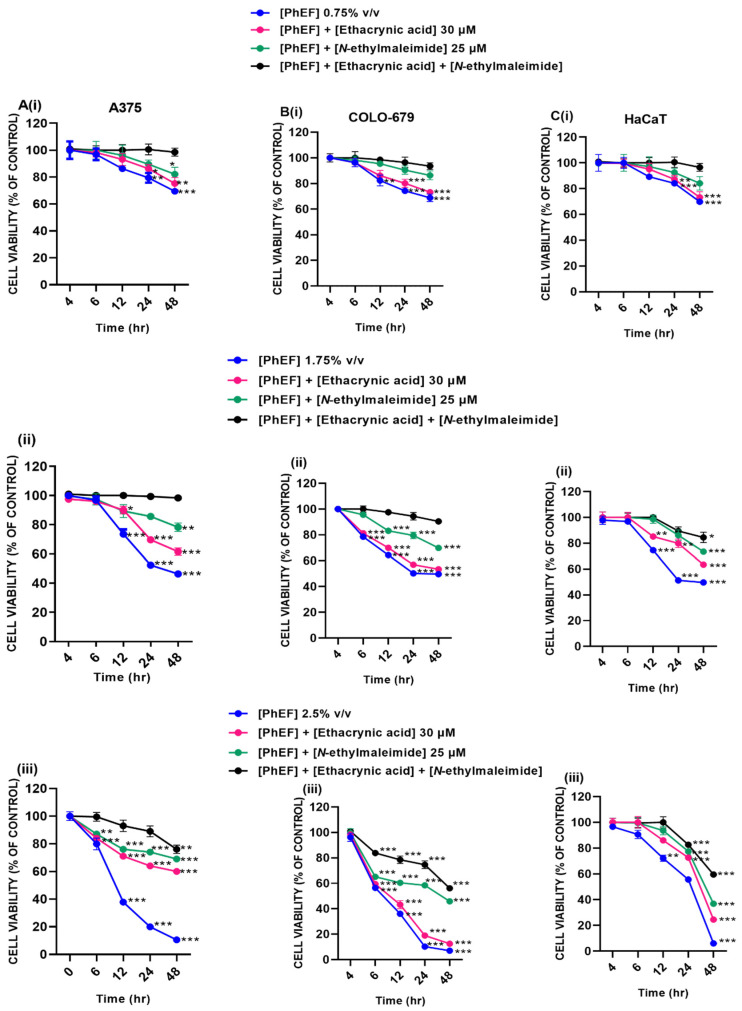
Cytotoxicity profiles of PhEF (0.75–2.5% *v*/*v*) alone or in combination with either ethacrynic acid (30 μM) or *N*-ethylmaleimide (25 μM) or both in A375 (**A**) (**i**)–(**iii**), COLO-679 (**B**) (**i**)–(**iii**) and HaCaT (**C**) (**i**)–(**iii**) cells over 4–48 h of exposure. Data are expressed as means ± SEM of three independent experiments. Statistical significance is indicated by * at *p* ≤ 0.05, ** at *p* ≤ 0.01 and *** at *p* < 0.001 relative to corresponding controls (DMSO 0.1% *v*/*v*).

To assess if PEITC or PEITC-NAC was responsible for the induction of cytotoxicity, A375 and COLO-679 cells were treated with either PhEF alone (0.75–2.5% *v*/*v*) or co-treated with ethacrynic acid (30 μM) or *N*-ethylmaleimide (25 μM) or both over 4–48 h (Figure 7).

Overall, results showed that PhEF can reduce cell viability levels in a time- and concentration-dependent manner (Figure 7). However, co-treatment with ethacrynic acid caused a marked increase in cell viability levels in all cell lines. The effect was more intense at the sub-toxic [Figure 7A(ii)–C(ii)] and toxic [Figure 7A(iii)–C(iii)] rather than the non-toxic [Figure 7A(i)–C(i)] concentrations of PhEF. Moreover, co-treatment with *N*-ethylmaleimide caused profoundly higher levels of cell viability at all PhEF concentrations, respectively [Figure 7A(i–iii)–C(i–iii)]. Finally, co-treatment of PhEF with ethacrynic acid and *N*-ethylmaleimide, caused an even further rescue in cell viability levels as they were even further increased in A375 and COLO-679 cells [Figure 7A(i,ii)–C(i,ii)] as compared to HaCaT cells where they were first rescued within 24 h followed with a marked reduction although at higher levels than any other exposure condition alone [Figure 7A(iii)–C(iii)].

## 4. Discussion

Previous studies have suggested that malignant melanoma cells are characterized by a dysregulated redox balance evident as higher basal ROS levels compared to non-tumorigenic keratinocytes and/or fibroblasts [41,42,43]. This can be attributed to ROS-induced melanoma progression and transformation, whereas ROS scavenging suppresses metastatic potency [44,45]. On the other hand, over-production of ROS can lead to apoptosis, autophagy, necrosis or ferroptosis [46,47,48,49]. PEITC has been shown, by others, to possess antioxidant properties by being capable of activating the nuclear factor erythroid 2–related factor-2/Kelch-like ECH-associated protein-1 (Nrf-2/KEAP-1) signaling pathway, thereby preventing the induction of oxidative stress [50,51,52]. In addition, our work has shown that PEITC can modulate the activity of key antioxidant enzymes associated with cellular protection against the generation of oxidative stress. For this reason, the activities of SOD, CAT, GR, GPx and GST were measured at non-toxic, sub-toxic and toxic concentrations of PhEF. Based on our results, GST activity was increased with increasing concentrations of PhEF, while the cellular concentration of GSH was decreased. Furthermore, GSH recycling was inhibited due to the inversely proportional relation between the PhEF concentration with GR and GPx. Consequently, the conjugation of PEITC with GSH has led to cellular GSH depletion and increased ROS production particularly at high concentrations of PhEF. On the other hand, the activity of SOD was increased, while that of CAT was inversely proportional to increasing PhEF concentrations, suggesting the capacity of both enzymes to metabolize ROS. However, other studies have shown that the inverse relationship between CAT and increased generation of ROS may be the result of CAT being reversibly inhibited or irreversibly deactivated or even degraded by ROS accumulation [53,54,55]. To this end, we have observed significant increases in lipid and protein oxidation statuses, as markers of increased ROS generation, at both the sub-toxic and toxic concentrations of PhEF. Our findings are in agreement with other studies demonstrating that cellular exposure to PEITC stimulates the oxidation of lipids, proteins and DNA [56,57]. Specifically, it has been hypothesized that lipid oxidation is a consequence of GSH depletion during PEITC metabolism, an effect also linked with the inability of GR and/or GPx to generate GSH [58,59]. However, when ROS scavengers were utilized (in combination with PEITC), they prevented lipid and/or protein oxidation, thereby highlighting the capacity of PEITC to induce ROS production [60]. 

Kinetic determination of PEITC metabolism (by monitoring the rate of PEITC-NAC adduct formation) has revealed, in the present study, that metabolism of sub-toxic and toxic concentrations of PhEF occurs rapidly within 6h and it is retained for up to 24 h. Furthermore, PEITC-NAC conjugate was found in the culture medium as early as 2 h post-treatment. Finally, we have demonstrated the direct involvement of GSH in PEITC metabolism since GSH levels were decreased as a function of time and PEITC concentration. To these ends, various in vivo models have demonstrated that the ingestion of ITCs leads to conjugation with GSH and subsequent excretion of ITC-NAC conjugate in urine [61]. Both unconjugated PEITC and PEITC-NAC conjugate have been shown to inhibit cancer cellular growth, thereby suggesting their use as potent chemo-preventing agents [62,63]. On the other hand, the co-administration of PhEF with ethacrynic acid (a GST inhibitor) induced rescue of cell viability, at all tested concentrations of PhEF, suggesting that the metabolic fate of PEITC is modulated by GST as its inhibition prevents GSH conjugation and depletion. On the contrary, NAT inhibition potentiated the observed rescue in cell viability, suggesting that the *N*-acetyl conjugate of PEITC plays a crucial role in inducing cytotoxicity. Finally, when inhibition of both GST and NAT was combined, cell viability levels were maintained at control levels, implying that PEITC-induced cytotoxicity was strongly associated with the formation of the *N*-acetylation by-product(s) as part of its metabolism. In addition, others have shown that the antineoplastic capacity of PEITC- and benzyl isothiocyanates (BITC)-NAC adducts, in A/J mice, was induced by the activation of MAP kinase cascade, increased the phosphorylation of p38 and increased the expression of extracellular signal-regulated (ErKs) 1 and 2 as well as c-Jun NH(2)-terminal kinases, thereby leading to increased apoptosis [28,63]. In another study, the inhibitory effect of PEITC and PEITC-NAC was demonstrated in a human hepatoma (SK-Hep1) cell line exhibiting a greater potency for NAC-PEITC (compared to PEITC) in inhibiting cancer cell adhesion, invasion and migration due to their ability to reduce the expression of matrix metalloproteinases (MMPs) -2, -9 and membrane type 1 (MT1) by increasing the expression of tissue inhibitors of matrix metalloproteinases (TIMPs) 1 and 2 [64]. Finally various studies have indicated that other ITCs including allyl (AITC) and sulforaphane (SFN) can selectively modulate the proliferation rate of pro-cancerous cells by increasing the activity of phase II xenobiotic-metabolizing enzymes, thus inducing cell cycle growth arrest or apoptosis [65,66]. 

## 5. Conclusions

To conclude, our findings provide an insight into the mechanism(s) by which ITCs induce cytotoxicity. Specifically, we have demonstrated that ITCs can form conjugates with GSH causing its depletion. Despite the increased activity of SOD, CAT was shown to be inactivated as the concentration of PhEF increased. On the other hand, GR and GPx appear not to be capable of utilizing GSH (for further redox cycling) as it is depleted (by conjugation with ITCs), thereby promoting the uncontrolled oxidation of lipids and proteins. Eventually, we have shown that PEITC-induced cytotoxicity is not attributed to the actual PEITC itself but rather to its *N*-acetyl conjugated form. Our findings enhance the current knowledge on how naturally derived PEITC can be utilized in adjuvant therapeutics protocols for the clinical management of malignant melanoma by means of enhancing the therapeutic potency and/or minimizing the toxicity of current clinical drugs.

## Figures and Tables

**Table 1:** Antioxidant activity of PhEF and PoEF as determined by the DPPH^●^, ABTS^●+^ and FRAP assays. Data are means ± SEM from three independent experiments with each experiment conducted in triplicates. Statistical significance is denoted by asterisks (***) at *p* ≤ 0.001. n.d. denotes “not determined”.

Sample	DPPH^●^	ABTS^●+^	FRAP
		IC_50_ (% *v*/*v*)	
PhEF	n.d.	n.d.	1.84 ± 0.2
PoEF	0.43 ± 0.03 ***	0.27 ± 0.01 ***	0.16 ± 0.01 ***

## Data Availability

Data are contained within the article.

## Supplementary Materials

The following supporting information can be downloaded at: https://www.mdpi.com/article/10.3390/antiox13010082/s1, Figure S1: Mass spectrum of the synthesized PEITC-NAC conjugate. Figure S2: ^1^H-NMR (A) and ^13^C-NMR (B) spectra of *N*_α_-acetyl-*S*-(*N*-phenethylthiocarbamoyl)-glutathione at 500 MHz and 100 MHz in DMSO-d*_6_* respectively. Table S1: Multiple reaction monitoring conditions for *N*_α_-acetyl-*S*-(*N*-phenethylthiocarbamoyl)-glutathione in UPLC-MS/MS analysis. Figure S3: Extracted UPLC-ESI-(+)-MS/MS chromatogram of *N*_α_-acetyl-*S*-(*N*-phenethylthiocarbamoyl)-glutathione. Figure S4: Calibration curve of *N*_α_-acetyl-*S*-(*N*-phenethylthiocarbamoyl)-glutathione standard at various concentrations (0.1–45 nM) used for the determination of the mercapturic acid end-product in intracellular space and culture medium. Table S2: The limit of detection (LOD), quantification (LOQ), linearity, precision and accuracy results for *N*_α_-acetyl-*S*-(*N*-phenethylthiocarbamoyl)-glutathione. The calibration equations represent the peak area as a function of concentration in nM. The intra- and inter-day experimental data have been collected over a six-day experiment. The % recovery data represent means of three independent experiments. Figure S5: Cytotoxicity profiles of A375 cells subjected to either ethacrynic acid (0–120 μM) (A) or *N*-ethylmaleimide (0–100 μM) (B) over 4–48 h of exposure. Data are expressed as means ± SEM of three independent experiments. n.d. represents data not determined. Statistical significance is indicated by ** at *p* < 0.01, *** at *p* < 0.001 relative to corresponding controls (DMSO 0.1% *v*/*v*). Table S3: Calculated EC_50_ values for ethacrynic acid and *N*-ethylmaleimide, in all cell lines, at different time points of exposure by utilizing an online EC_50_ calculator platform (Very Simple IC50 Tool Kit, available online: http://www.ic50.tk/ (accessed on 14 April 2023).
