# Supplementary material for: Naturally Derived Phenethyl Isothiocyanate Modulates Induction of Oxidative Stress via Its N-Acetylated Cysteine Conjugated form in Malignant Melanoma
Source: Antioxidants (Basel). 2024 Jan 8;13(1):82. doi: 10.3390/antiox13010082 (PMC10812449; doi:10.3390/antiox13010082)
Supplement: Supplementary file 1 [file antioxidants-13-00082-s001.zip › antioxidants-2795723-supplementary.pdf]

Supplementary Material

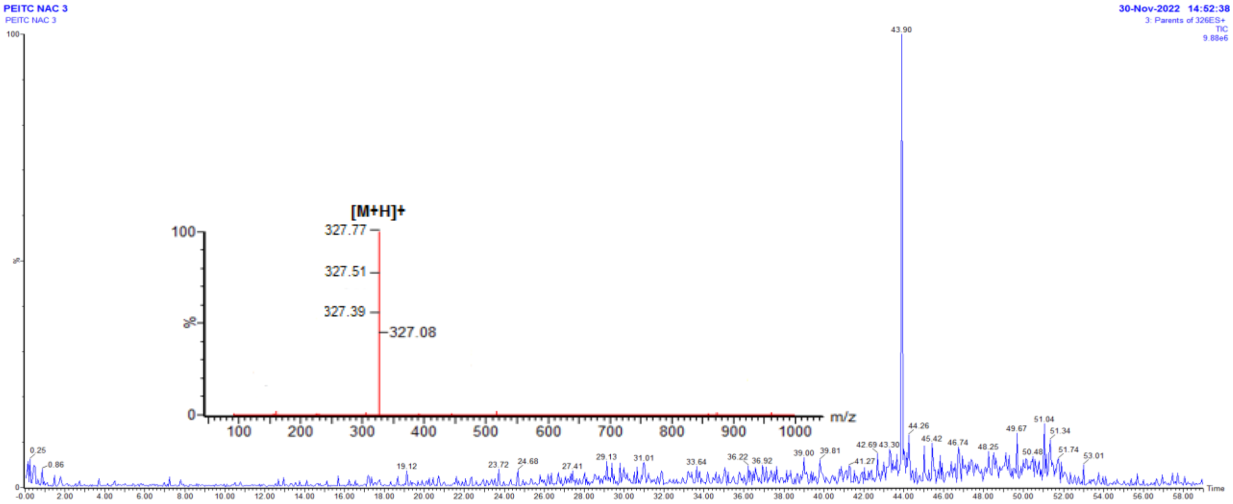

Figure S1: Mass spectrum of the synthesised PEITC-NAC conjugate.

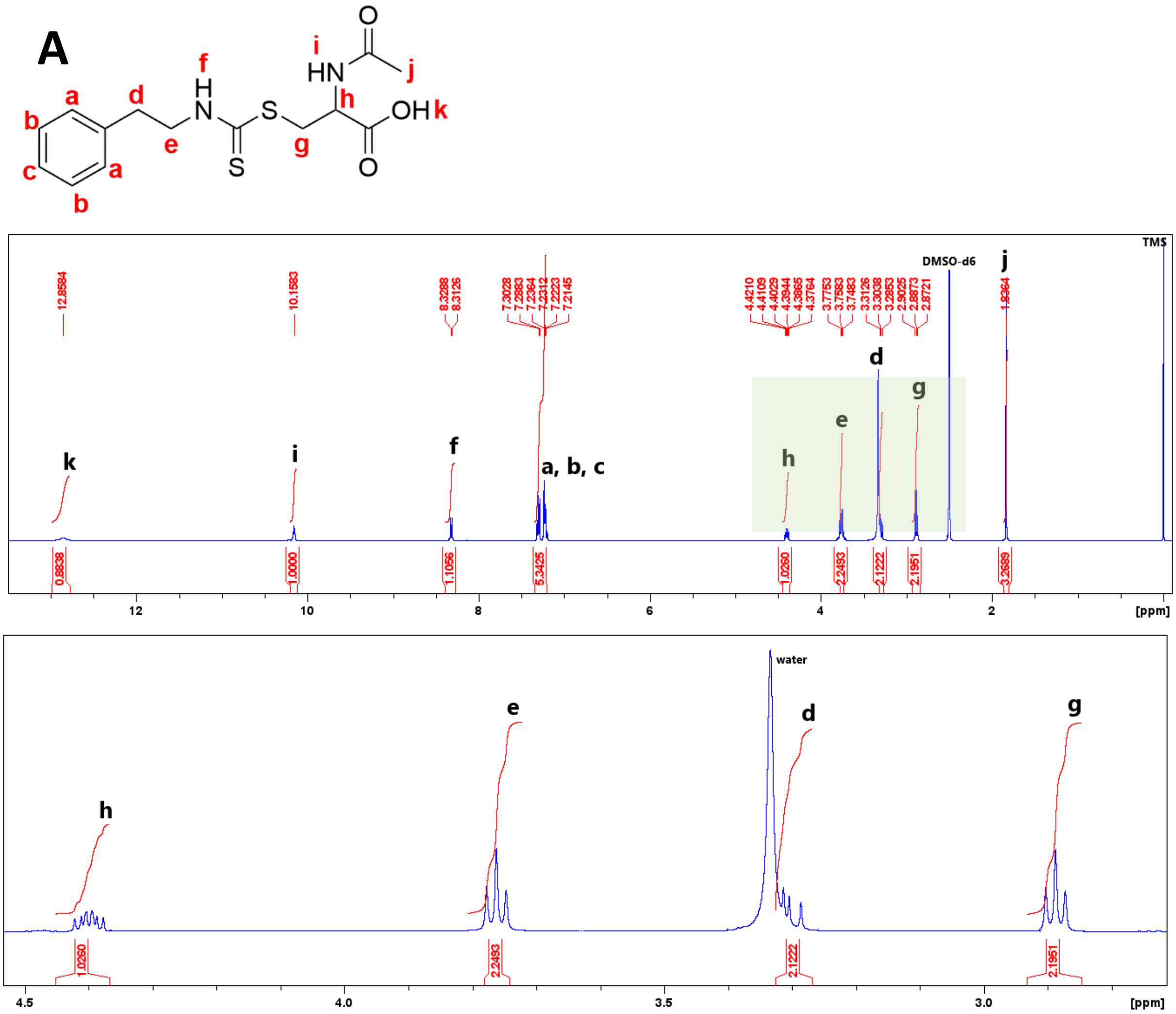

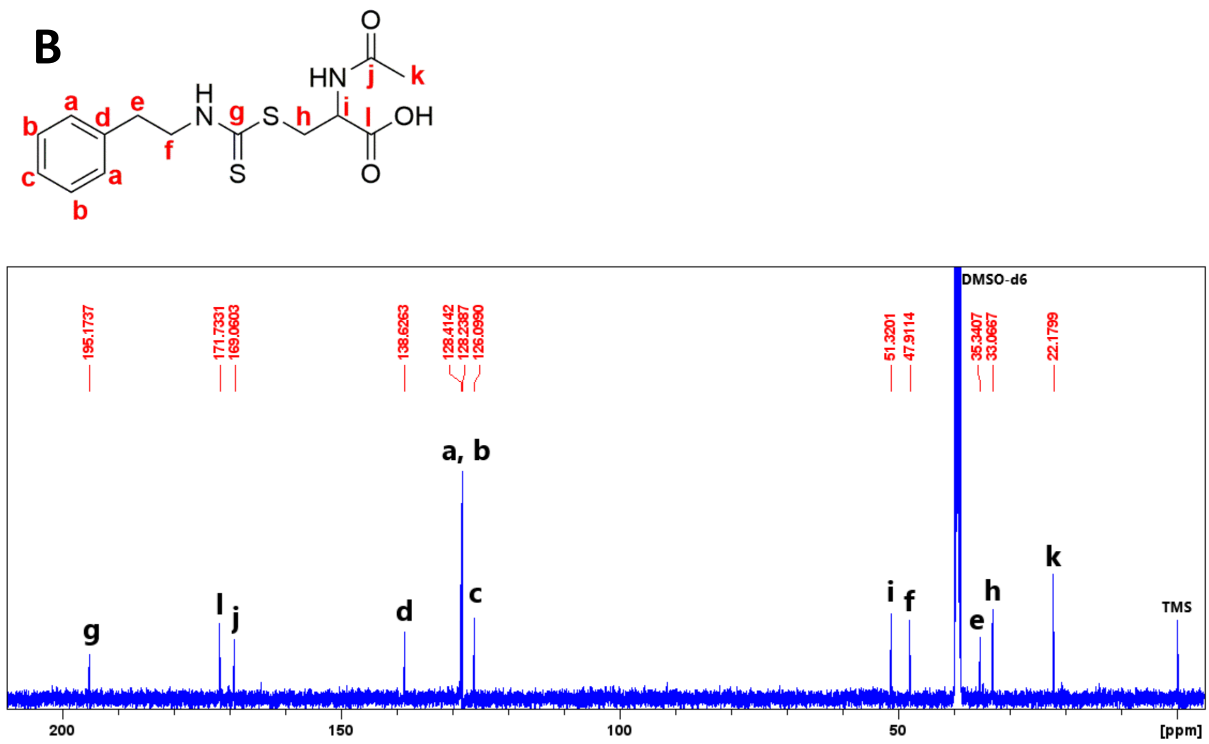

Figure S2: ^{1}H-NMR **(A)** and ^{13}C-NMR **(B)** spectra of *N*$_\alpha$-acetyl-*S*-(*N*-phenethylthiocarbamoyl)-glutathione at 500 MHz and 100 MHz in DMSO-d$_6$ respectively.

Table S1: Multiple reaction monitoring conditions for *N*$_\alpha$-acetyl-*S*-(*N*-phenethylthiocarbamoyl)-glutathione in UPLC-MS/MS analysis

**Compound**	**Chemical formula**	**MW**	**[M-H]$^+$ (m/z)**	**MS^2 fragments (m/z)**	**Cone voltage (V)**	**Collision energy (eV)**	**Retention time (R$_t$) (min)**
N$_\alpha$-acetyl-*S*-(*N*-phenethylthiocarbamoyl)-glutathione	$C_{14}H_{18}N_2O_3S_2$	326.43	327.77	197.42	15	23	43.9

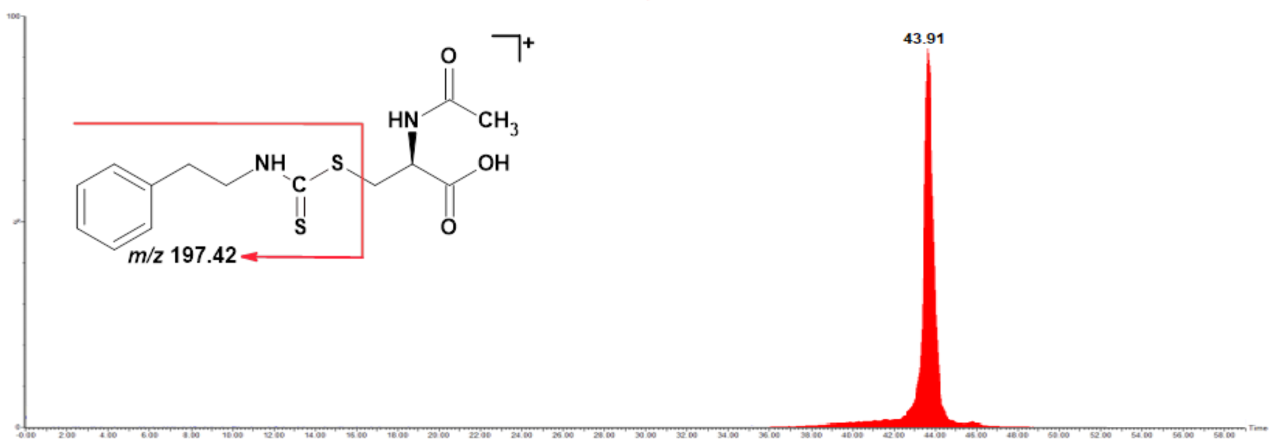

Figure S3: Extracted UPLC- ESI-(+)-MS/MS chromatogram of N_α-acetyl-*S*-(*N*-phenethylthiocarbamoyl)-glutathione.

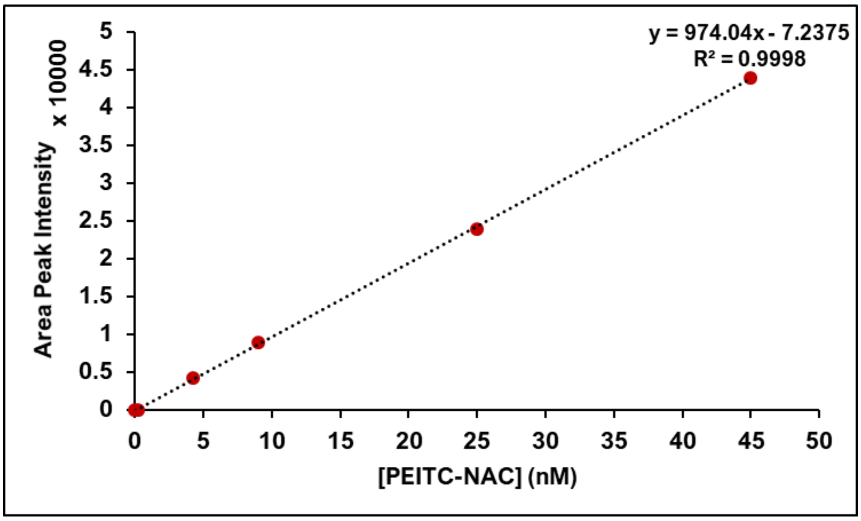

Figure S4: Calibration curve of N_α-acetyl-*S*-(*N*-phenethylthiocarbamoyl)-glutathione standard at various concentrations (0.1-45 nM) used for the determination of the mercapturic acid end-product in intracellular space and culture medium.

Table S2: The limit of detection (LOD), quantification (LOQ), linearity, precision and accuracy results for N_α-acetyl-*S*-(*N*-phenethylthiocarbamoyl)-glutathione. The calibration equations represent the peak area as a function of concentration in nM. The intra- and inter- day experimental data have been collected over a six days experiment. The % recovery data represent means of three independed experiments.

Compound	Linear range (nM)	*LoD* (nM)	*LoQ* (nM)	Calibration equation[a]	Correlation coefficient (r^2)	%RSD (intra-day)[b]	%RSD (inter-day)[c]	%REC[d]
N_α-acetyl-*S*-(*N*-phenethylthiocarbamoyl)-glutathione	0.21-45	0.21	6.93	y = 974.04x-7.235	0.9998	0.96	2.56	98.8

[a] Chromatographic peak area (y) as a function of nM concentration (x)

[b] Values are means of intra-day assays (*n*=6)

[c] Values are means of inter-day assays (*n*=6)

[d] (*n*=3)

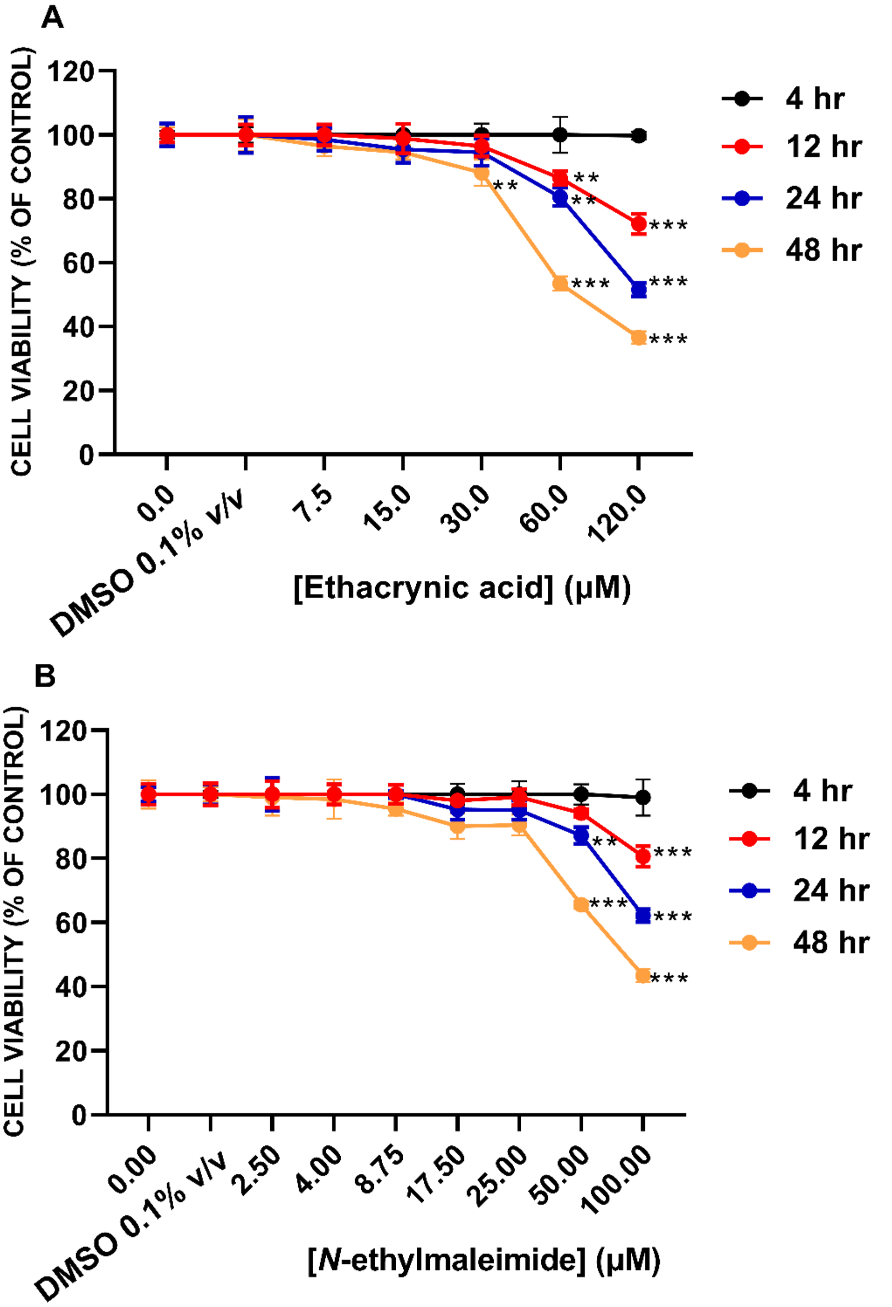

Figure S5: Cytotoxicity profiles of A375 cells subjected to either ethacrynic acid (0-120 µM) (**A**) or N-ethylmaleimide (0- 100 µM) (**B**) over 4-48 hr of exposure. Data are expressed as means ± SEM of three independed experiments. *n.d.* represents data not determined. Statistical significance is indicated by **at p<0.01, ***at p<0.001 relative to corresponding controls (DMSO 0.1% v/v).

Table S3: Calculated EC50 values for ethacrynic acid and *N*-ethylmaleimide, in all cell lines, at different time point of exposure by utilizing an online EC_{50} calculator platform (Very Simple IC50 Tool Kit, available online: http://www.ic50.tk/ (accessed on 14 April 2023).

**Time (hr)**	**Ethacrynic acid**	***N*-ethylmaleimide**
	**EC50 (μM)**	
4	n.d.	n.d.
12	n.d.	n.d.
24	114.98±4.21	n.d.
48	55.48±1.25	83.56±2.64
